# MEK-Inhibitor-assoziierte Retinopathie unter Binimetinib-Therapie bei kutanem malignem Melanom

**DOI:** 10.1007/s00347-020-01089-3

**Published:** 2020-04-04

**Authors:** S. Groselli, D. Heinrich, C. P. Lohmann, M. Maier

**Affiliations:** Klinik und Poliklinik für Augenheilkunde, Klinkum rechts der Isar, Technischen Universität München, Ismaninger Str. 22, 81675 München, Deutschland

**Keywords:** BRAF-Hemmer, Nebenwirkungen, Retinopathie, Optische Kohärenztomographie, Neurosensorische Netzhautabhebung, BRAF inhibitor, Side effects, Retinopathy, Optical coherence tomography, Neurosensory retinal detachment

## Abstract

Die Behandlungsmöglichkeiten für Patienten mit metastasierendem Melanom (MM) wurden in den letzten Jahren mit der Zulassung von neuen Medikamenten dramatisch erweitert. Die MEK(mitogen-aktivierte Proteinkinase-Kinasen)- und BRAF(Serin/Threonin-Kinase B-Raf kodierendes Gen)-Hemmer-Kombinationstherapie gehört aktuell zum Versorgungsstandard für das Stadium IIIC/IV des BRAF-mutierten Melanoms. MEKAR (MEK-Inhibitor-assoziierte Retinopathie) werden bei Patienten mit metastasierendem Melanom beobachtet, die mit einer solchen Kombinationstherapie behandelt werden bzw. wurden. Wir berichten über den Fall eines 72-jährigen Patienten, der eine solche Pathologie unter der Therapie mit Binimetinib in Kombination mit Nivolumab erlitt. Diese Kasuistik verdeutlicht die Wichtigkeit einer interdisziplinären Zusammenarbeit bei der Behandlung von MM-Patienten.

## Abstract (en)

The treatment options for patients with metastatic melanoma (MM) have been dramatically expanded in recent years with the approval of new drugs. The MEK (mitogen-acitvated protein kinase kinase) and BRAF (serine/threonine-protein kinase B-Raf coding gene) inhibitor combination therapy is currently part of the standard of care for stage IIIC/IV of BRAF mutant melanoma. The MEK inhibitor-associated retinopathy (MEKAR) is observed in patients with MM who are treated (or have been treated) with such a combination therapy. This article reports the case of a 72-year-old male patient, who suffered from such a pathological condition under treatment with binimetinib in combination with nivolumab. This case study illustrates the importance of interdisciplinary collaboration in the treatment of MM patients.

## Falldarstellung

### Anamnese

Ein 72-jähriger männlicher Patient wurde uns durch den niedergelassenen Augenarzt notfallmäßig zur Mitbeurteilung einer Visusminderung bei Verdacht auf eine beidseitige zentrale seröse Chorioretinopathie (CSCR) überwiesen.

### Klinischer Befund

Unsere Untersuchungen zeigten bei einem bestkorrigierten Visus von 0,4 (+2,0 −1,0 85°) am rechten Auge und 0,9 (+1,5 −0,75 125°) am linken Auge reizfreie vordere Augenabschnitte mit beidseits inzipienter Katarakt. Der Augeninnendruck war normoton. Fundoskopisch imponierten an beiden Augen helle, teils prominente Areale in allen Quadranten. Die Papille war randscharf und vital gefärbt. Die Gefäße waren unauffällig.

### Diagnostik

**Figure 1:**
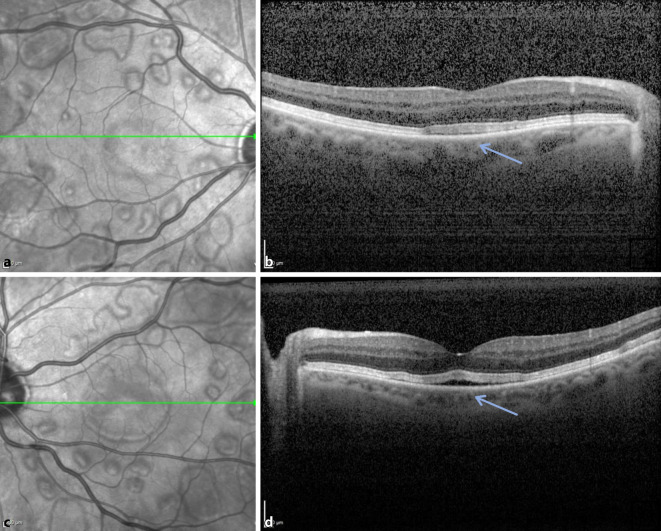

Wir führten eine Spectral-Domain-optische Kohärenztomographie (SD-OCT) durch, in der wir multiple umschriebene neurosensorische Netzhautabhebungen sowohl im Bereich der Makula wie auch in der Peripherie feststellen konnten. Das retinale Pigmentepithel (RPE) und die Aderhaut waren nicht betroffen. An der Makula konnten wir ebenfalls eine seröse Flüssigkeitsansammlung zwischen RPE und den Fotorezeptoraußensegmenten feststellen (Abb. 1). Wir vermuteten eine MEK (mitogen-aktivierte Proteinkinase-Kinasen)-Inhibitor-assoziierte Retinopathie (MEKAR).

**Figure 2:**
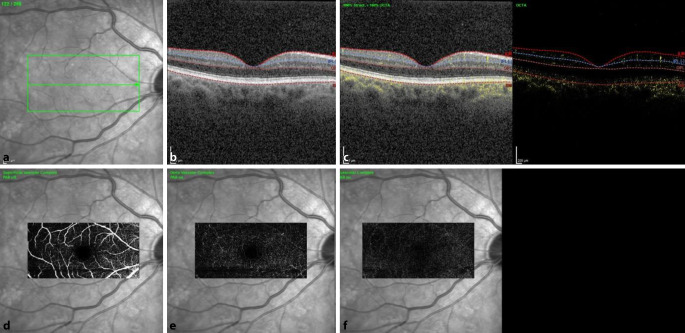

**Figure 3:**
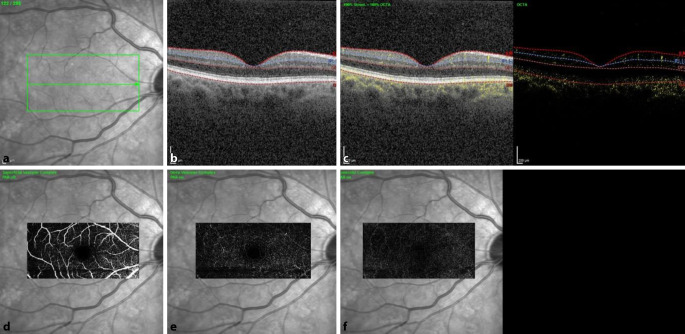

**Figure 4:**
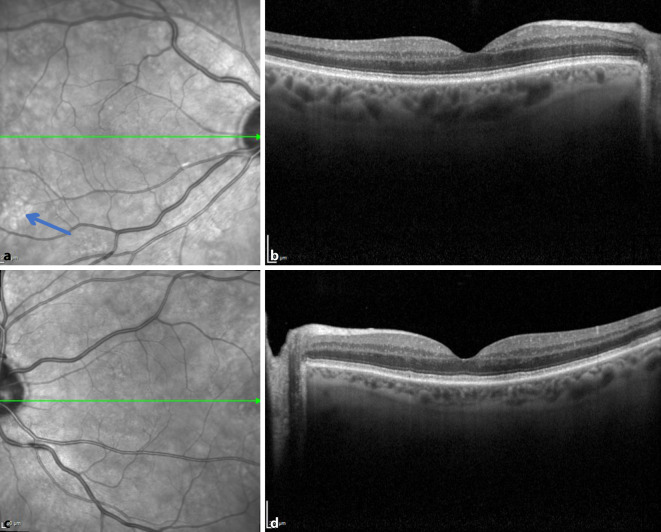

### Therapie und Verlauf

In Zusammenschau sämtlicher Befunde gingen wir von einer MEK-Inhibitor-assoziierten Retinopathie im Rahmen einer Binimetinib-Therapie bei malignem Melanom aus.

Eine Woche nach Erstvorstellung bestellten wir den Patienten erneut in unsere Sprechstunde ein. Der Visus war beidseits unverändert, der Patient berichtete jedoch von einer Symptomverbesserung. Wir führten eine erneute SD-OCT-Aufnahme und eine OCT‑A (optische Kohärenztomographie – Angiographie) durch. In der SD-OCT konnte man beidseits Pigmentepithelunregelmäßigkeiten, jedoch keine intra- oder subretinale Flüssigkeit mehr feststellen. Die foveale Depression war an beiden Augen erhalten. In der OCT-A-Aufnahme zeigte sich beidseits eine regelrechte Gefäßdarstellung (Abb. 2 und 3). Fundoskopisch konnte man bereits eine Regression der hellen Areale feststellen. Der Patient teilte uns mit, dass man nun aufgrund der zahlreich aufgetretenen Nebenwirkungen die Therapie mit Binimetinib einstellen wolle.

Zwei Wochen nach Erstvorstellung war anamnestisch eine Visusverbesserung zu erheben, auch die Kreise und die schwarzen Flecke wurden nun von unserem Patienten nicht mehr bemerkt. Der Visus betrug am rechten Auge bestkorrigiert 0,6 und am linken 0,7. Fundoskopisch und in der OCT konnte man keine neurosensorische Abhebungen mehr feststellen (Abb. 4). Es waren lediglich einige wenige hyperreflektive Areale zu sehen (blauer Pfeil Abb. 4a). Der Patient berichtete nun, dass man die Therapie aufgrund der zahlreichen Nebenwirkungen pausiert habe.

## Diskussion

UV-Strahlung gilt als der bedeutsamste Risikofaktor in der Ätiologie von Hautkrebs. Es gibt zahlreiche Hinweise, dass das maligne Melanom aufgrund intermittierender UV-Exposition und schwerer Sonnenbrände in der Kindheit und Jugend auftritt. Für das maligne Melanom gibt es außerdem Hinweise, dass es autosomal-dominant vererbbar ist, da 5–12 % der erkrankten Patienten einen oder mehrere Verwandte 1. Grades aufweisen, die ebenfalls am malignen Melanom erkrankt sind. Als mögliches Vorläuferstadium des malignen Melanoms werden benigne Nävi angenommen. Es wird vermutet, dass diese durch klonale Proliferation aus Melanozyten entstanden sind, die wahrscheinlich aufgrund zellulärer Seneszenz die Proliferation einstellen. Diese Wachstumsinhibition kann z. B. durch RAS-Mutationen oder durch Mutationen im *BRAF*-Gen aufgehoben werden. Dies kann zur Bildung dysplastischer Nävi und nachfolgend zur radialen Wachstumsphase des malignen Melanoms führen [8].

Die Inzidenz des malignen Melanoms nimmt am schnellsten unter allen Krebsarten zu [3]. Aktuell erkranken in Deutschland jährlich rund 18.000 Personen an einem invasiven malignen Melanom, hiervon sind 51,5 % männlichen Geschlechts. Bei Männern ist das MM die achthäufigste und bei Frauen die vierthäufigste Krebsneuerkrankung. Die Erkrankungshäufigkeit steigt mit zunehmendem Alter an. Junge Frauen erkranken häufiger als junge Männer an einem MM. Ab dem Alter von 60 Jahren kehrt sich das Verhältnis jedoch um, und die Inzidenz bei Männern steigt auf das 2‑Fache der Inzidenz bei Frauen an [8]. Beim Stellen der Diagnose sind bei etwa 2–5 % der Patienten bereits Metastasen vorhanden [3]. Häufig findet man Metastasen in der Leber, in der Haut, in der Lunge, im Skelett oder im Gehirn.

Die Behandlungsmöglichkeiten für Patienten mit metastasierendem Melanom, insbesondere dem BRAF-mutierten-Melanom, wurden in den letzten Jahren mit der Zulassung von neuen Medikamenten dramatisch erweitert. Der auf BRAF ausgerichtete Standardansatz hat sich von der Einzelwirkungshemmung von BRAF auf die Kombinationstherapie mit einem BRAF- und einem MEK-Inhibitor verlagert [6]. MEK- und BRAF-Hemmer sind neuere Gruppen von Medikamenten, die auf die Targetenzyme des MAPK/ERK-Signalwegs wirken. Diese Kombinationstherapie gehört aktuell zum Versorgungsstandard für das Stadium IIIC/IV des BRAF-mutierten Melanoms [3]. Gleichzeitig ist die Immuntherapie von der zytokinbasierten zur antikörpervermittelten Behandlung übergegangen. Diese Veränderungen in den Therapieansätzen haben die Patientenergebnisse drastisch verbessert, wobei sich die mediane Gesamtüberlebenszeit von Patienten mit Melanom im fortgeschrittenen Stadium von etwa 9 Monaten vor 2011 auf mindestens 2 Jahre erhöht hat [6, 7].

MEKAR (MEK-Inhibitor assoziierte Retinopathie) werden bei Patienten mit metastasierendem Melanom beobachtet, die mit MEK-Inhibitor und/oder in Kombination mit BRAF-Inhibitor behandelt werden bzw. wurden. Präklinische Studien zeigten, dass eine MEK-Hemmung zu einer akuten RPE-Toxizität führt, die zu einer RPE-Hyperpermeabilität und zum Abbau der Netzhaut-Blut-Schranke führt. Die Läsionen sind entweder solitäre oder multiple bilaterale seröse neurosensorische Netzhautablösungen. Fokale Läsionen sind v. a. in den äußeren Netzhautschichten bemerkbar [3]. Es ist denkbar, dass Schwankungen systemischer Zytokinspiegel im Rahmen der Tumorabwehr zu serösen Retinopathien beitragen. Am ehesten ist jedoch laut Grajewski et al. von einem multifaktoriellen Prozess auszugehen, in dem Hormone, Mediatoren und Inhibitoren zusammenwirken, die solche Retinopathien hervorrufen könnten [1].

MEKAR-Läsionen können mit nichtinvasiven diagnostischen Bildgebungsmethoden wie OCT- und IR-Bildgebung erkannt und von serösen Netzhautablösungen anderer Genese unterschieden werden. Die Aderhautdicke ist bei MEKAR normal. In den OCT-Aufnahmen ist hyperreflektive SRF unter der Interdigitalzone (Sub-IR) bemerkbar [3]. Die extrafovealen Läsionen befinden sich meist um die Gefäßbögen. Es wird vermutet, dass dies aufgrund der höheren Konzentration des Medikaments in größeren Gefäßen geschieht [4]. Bei unserem Patienten zeigten sich multiple neurosensorische Abhebungen ohne Veränderungen in der OCT‑A. Ein ähnlicher Fall wurde auch von Lüdeke et al. beschrieben, bei dem in der durchgeführten Fluoreszenzangiographie keine Leckage darstellbar war, weshalb der Ursprung der subretinalen Flüssigkeit bislang unklar bleibt [5].

Auch Grajewski et al. beschrieben 2 ähnliche Fälle von Patienten mit metastasiertem kutanem malignem Melanom, bei denen multifokale bilaterale Netzhautläsionen in Form von serösen Ablösungen und vitelliformen Ablagerungen auftraten. Bei beiden Patienten ähnelten die Läsionen in den SD-OCT-Befunden denen einer zentralen serösen Chorioretinopathie (CRCS). Auch die pseudovitelliformen Ablagerungen wurden bei CRCS bereits beschrieben. Die Ätiologie der CRCS ist zwar unbekannt, allerdings wurde bereits mehrmals beobachtet, dass ein erhöhter Kortikosteroidspiegel im Serum einen negativen Einfluss auf das klinische Erscheinungsbild haben kann. Bei Patienten mit kutanem Melanom gibt es mehrere Berichte über eine ektopische paraneoplastische Produktion von adrenokortikotropem Hormon (ACTH), das zu einem erhöhten Kortisolserumspiegel führt. Daher wurde bei dem von Grajewski et al. dargestellten Fall ein Dexamethason-Suppressionstest durchgeführt, um einen evtl. endogenen Hyperkortisolismus auszuschließen. Die endokrinologischen Parameter zeigten sich innerhalb des Normalbereichs. Da dieser Test jedoch 4 Jahre nach dem Auftreten der CRCS-ähnlichen Läsionen durchgeführt wurde, ist es nicht möglich, frühere Episoden von ektopischer ACTH-Produktion bei diesem Patienten sicher auszuschließen [2].

In einer durch Francis et al. durchgeführten Studie berichteten 12 Patienten (48 %) zum Zeitpunkt der Ansammlung der SRF von Symptomen, die denen einer CRCS ähneln [4]. Es wurde berichtet, dass bei Patienten, die mit dem MEK-Hemmer Binimetinib behandelt wurden, insbesondere in den ersten 4 Wochen der Behandlung Sehstörungen auftreten. Befindet sich die Läsion in der Foveola, können die Patienten Symptome wie einen Kreis zentral im Gesichtsfeld oder verschwommenes Nahsehen beschreiben [3]. Während der Entwicklungsphase verschiedener MEK-Inhibitoren erkannte man das toxische Potenzial für die Augen insbesondere im Sinne retinaler Venenverschlüsse und einer der CRCS ähnlichen serösen Retinopathie. Beschriebene Symptome waren unter anderem Halos, Photophobie, Diplopie und Epiphora [5]. Weitere allgemeine Nebenwirkungen von MEK-Inhibitoren sind Obstipation, Übelkeit, Erbrechen, Kopfschmerzen und Schwindelgefühl.

## Fazit für die Praxis

- Augenärzte und Onkologen müssen sich dieser häufigen, aber relativ harmlosen und oft vorübergehenden Nebenwirkung der Behandlung mit MEK-Inhibitor in Kombination mit BRAF-Inhibitor bewusst sein, um unnötige Eingriffe zu vermeiden, einschließlich des Absetzens einer möglicherweise lebensverlängernden Therapie.
- Hierbei ist eine interdisziplinäre Zusammenarbeit zwischen Onkologen, Dermatologen und Augenärzten bei der Behandlung von MM-Patienten hilfreich.
- Empfehlenswert wäre beispielsweise eine gezielte Untersuchung von MM-Patienten vor und während des Therapiezeitraumes durch den Augenarzt, um den Patienten bestmöglich über die evtl. vorübergehenden Nebenwirkungen der Therapie aufzuklären und während des Behandlungszeitraumes zu begleiten.
